# Blastocyst quality and congenital malformation risk in singleton births after frozen embryo transfer

**DOI:** 10.1038/s41598-025-20150-2

**Published:** 2025-10-17

**Authors:** Linqing Du, Tian Ye, Wenqian Fan, Huijuan Kong

**Affiliations:** 1 Center for Reproductive Medicine, The First Affiliated Hospital of Zhengzhou University, No.1, Jianshe Road, Zhengzhou, China; 2 Henan Key Laboratory of Reproduction and Genetics, The First Affiliated Hospital of Zhengzhou University, Zhengzhou, China; 3 Henan Provincial Obstetrical and Gynecological Diseases (Reproductive Medicine) Clinical Research Center, The First Affiliated Hospital of Zhengzhou University, Zhengzhou, China; 4 Henan Engineering Laboratory of Preimplantation Genetic Diagnosis and Screening, The First Affiliated Hospital of Zhengzhou University, Zhengzhou, China

**Keywords:** Blastocyst quality, Congenital malformation, Frozen embryo transfer, Neonatal outcomes, Obstetric complications, Endocrinology, Medical research

## Abstract

The relationship between blastocyst morphological quality and the risk of congenital malformations in assisted reproductive technology (ART) remains poorly understood, limiting clinical decision-making for embryo selection. We conducted a retrospective cohort study of 3986 frozen embryo transfer cycles (January 2014–June 2023) to evaluate whether blastocyst morphological quality influences the risk of congenital malformations. Blastocysts were classified according to Gardner’s grading system, and 1:2 propensity score matching was applied to control for maternal age, BMI, infertility characteristics, and other potential confounders. After matching, 1743 singleton births were analyzed (1162 good-quality vs. 581 poor-quality blastocysts). Baseline characteristics were well balanced between groups. The risk of congenital malformations was similar between good- and poor-quality groups (aOR 1.14, 95% CI 0.54–2.41, *P* = 0.7310; 1.72% vs. 2.07%), with no significant between-group differences in any ICD-10 organ-specific categories (all P > 0.10). Secondary outcomes showed no significant differences: preterm birth (aOR 0.80, 95% CI 0.57–1.12, *P* = 0.1976), low birth weight (aOR 1.26, 95% CI 0.72–2.19, *P* = 0.4172), other neonatal outcomes, and obstetric complications (aOR 0.89, 95% CI 0.64–1.22, *P* = 0.4629). These findings indicate that blastocyst morphological quality does not influence the risk of congenital malformations, supporting the use of morphologically poor blastocysts when high-quality alternatives are unavailable, which may reduce unnecessary discarding of embryos, alleviate patient anxiety, and improve treatment accessibility.

**Supplementary Information:**

The online version contains supplementary material available at 10.1038/s41598-025-20150-2.

## Introduction

Assisted reproductive technology (ART) has transformed the landscape of infertility treatment worldwide, with frozen-thawed embryo transfer (FET) cycles emerging as the predominant approach in modern fertility practice^1,2^. Despite progress in time‑lapse imaging and AI‑assisted evaluation, morphological assessment continues to serve as the cornerstone of embryo selection in IVF laboratories globally^3,4^.

The clinical implications of transferring morphologically poor-quality embryos have garnered increasing attention, particularly concerning potential birth defects. This issue has become increasingly pertinent as approximately 30–40% of available embryos are classified as poor quality according to standard morphological criteria^5,6^. The decision-making process becomes especially challenging in scenarios of limited embryo availability or within jurisdictions imposing regulatory restrictions on embryo creation^7^.

The relationship between embryo quality and congenital malformation risk remains controversial in current literature. Systematic reviews and meta-analyses have produced conflicting results, with some investigations suggesting increased risks associated with poor-quality embryo transfers^8,9^, while others demonstrate comparable safety profiles between poor- and good-quality embryos^10,11^. These disparate findings may be attributed to substantial methodological heterogeneity across studies, including variations in embryo quality classification systems and insufficient control for potential confounding factors^12^.

The interpretation of existing research is further complicated by several methodological limitations. Current evidence is constrained by non-standardized embryo grading systems, limited statistical power for detecting rare congenital anomalies, and the conflation of fresh and frozen-thawed cycle outcomes. Moreover, inadequate adjustment for critical parental factors and insufficient long-term developmental follow-up have undermined the reliability of available data^13^. These methodological challenges underscore the necessity for more rigorously designed investigations.

Therefore, this study aimed to investigate the association between embryo quality and congenital malformation risk in frozen-thawed blastocyst transfer cycles.

## Materials and methods

### Study design and population

This retrospective cohort study was conducted at the Reproductive Medical Center of the First Affiliated Hospital of Zhengzhou University between January 2014 and June 2023. For each patient, only the first FET cycle with single blastocyst transfer that resulted in a singleton live birth was included. We excluded cycles involving donor sperm or oocytes, chromosomal abnormalities, endometriosis, Müllerian duct anomalies, endocrine disorders (including diabetes and thyroid disorders), or preimplantation genetic testing (PGT). The study protocol was approved by the Ethics Committee of the First Affiliated Hospital of Zhengzhou University (approval number: 2024-KY-1203-001). The study design and implementation followed the strengthening the reporting of observational studies in epidemiology (STROBE) guidelines^14^.

### Blastocyst evaluation and group assignment

Blastocysts were graded according to the Gardner system^15^. Based on the 2022 Chinese Expert Consensus on Human Cleavage-stage Embryo and Blastocyst Morphological Evaluation^16^, good-quality blastocysts were defined as those achieving ≥ grade 3 expansion with inner cell mass and trophectoderm grades of AA, AB, BA, or BB on day 5, or ≥ grade 4 expansion with the same quality grades on day 6. All other blastocysts were classified as poor quality. Following these criteria, 3023 transfers were classified into the good-quality group and 963 into the poor-quality group.

### Outcome measures and data collection

The primary outcome was congenital malformations, identified and classified according to the International Classification of Diseases, Tenth Revision (ICD-10; codes Q00–Q99)^17^. Secondary outcomes included neonatal and obstetric outcomes. For neonatal outcomes, birth weight categories followed WHO standards^18^: low birth weight (LBW, < 2500 g), very low birth weight (VLBW, < 1500 g) and high birth weight (HBW, ≥ 4000 g). Fetal growth centiles were derived from the sex- and gestational age–specific Chinese birth weight-for-gestational-age reference curves^19^, and neonates were classified as small for gestational age (SGA, < 10th percentile), very SGA (VSGA, < 3rd percentile), large for gestational age (LGA, ≥ 90th percentile) and very LGA (VLGA, ≥ 97th percentile). NICU admission was defined as any transfer to a neonatal intensive care unit after birth.

For obstetric outcomes, gestational age was estimated by imputing the last menstrual period (LMP) as 14 days plus embryo age before the transfer date. Mode of delivery was categorized as spontaneous vaginal delivery or cesarean section. Preterm birth was defined as delivery before 37 completed weeks of gestation. Obstetric complications included premature rupture of membranes (PROM; rupture of membranes before the onset of labor); stillbirth (fetal death at ≥ 20 weeks’ gestation); placenta previa; gestational hypertension; gestational diabetes mellitus; cervical insufficiency; fetal distress; and amniotic fluid abnormalities, including oligohydramnios and polyhydramnios.

Clinical and laboratory data were retrospectively extracted from the electronic medical record (EMR). EMR variables included demographic characteristics (age, body mass index), infertility-related parameters (primary/secondary infertility, infertility factors, duration), treatment-related parameters (fertilization method, endometrial preparation protocol, embryo development day). For all EMR variables, we used the values documented on the day of embryo transfer. Follow-up data were collected through standardized telephone interviews conducted by trained nurses and included pregnancy complications, delivery details, neonatal outcomes, and congenital malformations.

### Statistical analysis

To minimize selection bias, propensity score matching was performed^20^. The propensity score model included maternal age, body mass index, infertility type, fertilization method, endometrial preparation protocol, and embryo development day. A 1:2 nearest-neighbor matching algorithm with a caliper width of 0.05 standard deviations was applied. Continuous variables were presented as mean ± standard deviation (SD) or median [Q1–Q3], as appropriate, and categorical variables were presented as counts and percentages. Within-group normality was assessed using the Shapiro–Wilk test together with Q–Q plots, and homogeneity of variances was assessed using Brown–Forsythe (Levene-type) test. Summary results of normality and variance homogeneity tests are provided in Supplementary Table S1, and Q–Q plots with histograms are shown in Supplementary Fig. S1. Based on these diagnostics, Welch’s t-test was used for approximately normal data and/or unequal variances, and the Wilcoxon rank-sum test for skewed distributions; categorical variables were compared using the chi-square test or Fisher’s exact test, as appropriate. Multivariate logistic regression analysis was used to evaluate the association between embryo quality and perinatal outcomes, with results presented as adjusted odds ratios (aORs) with 95% confidence intervals (CIs). Statistical analyses were performed using EmpowerStats (EmpowerROS; X&Y Solutions, Inc., Boston, MA, USA), version 4.2 (https://www.empowerstats.com), which is based on R (version 4.3.1; https://www.r-project.org). Two-sided *P *values < 0.05 were considered statistically significant.

## Results

**Fig. 1:**
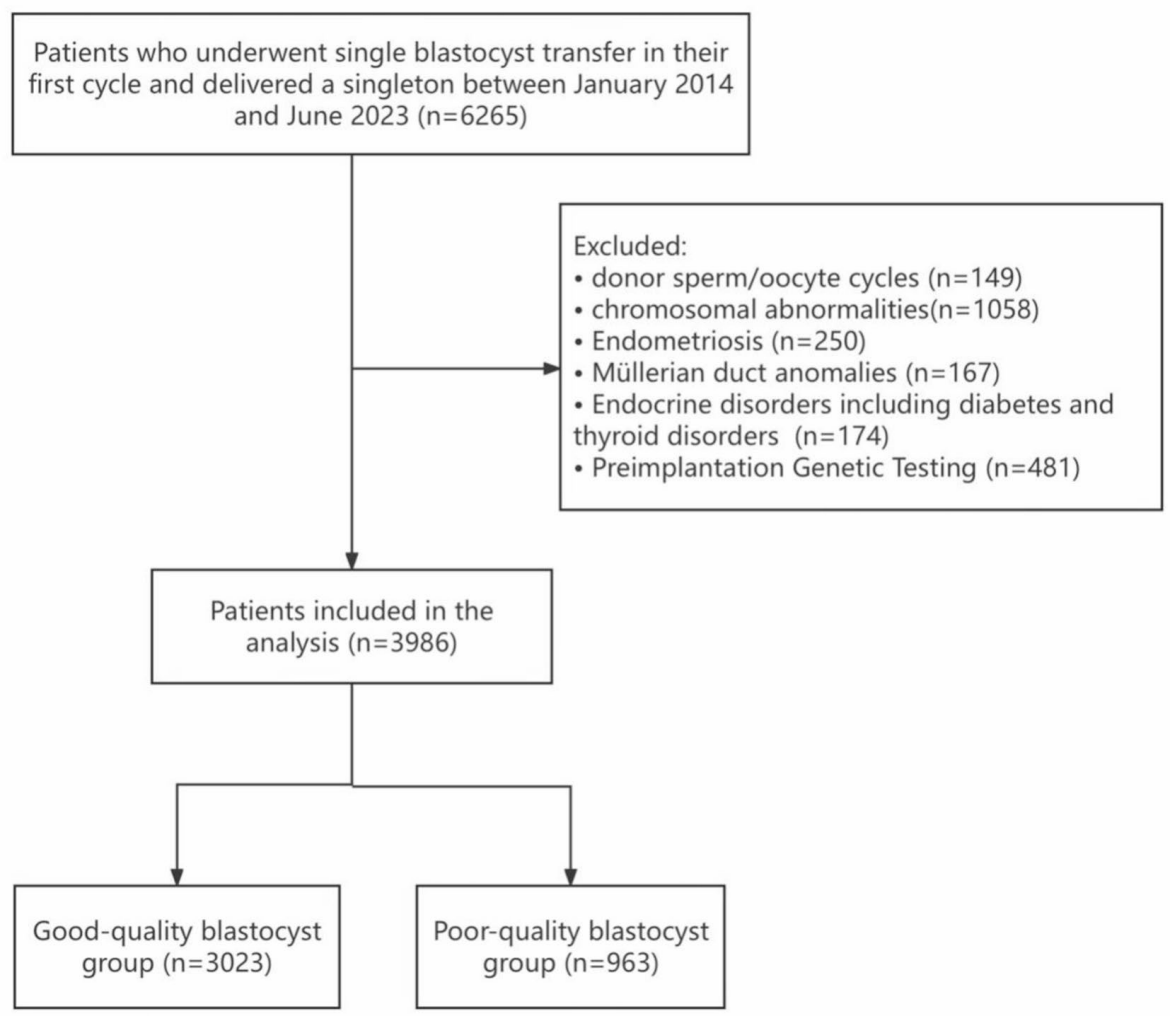
Flowchart of study population and embryo quality grouping.

As shown in Fig. 1, a total of 6265 patients who underwent single blastocyst transfer in their first cycle and delivered a singleton during the study period were initially screened. Patients were excluded if they met any of the following criteria: donor sperm/oocyte cycles (n = 149), chromosomal abnormalities (n = 1058), endometriosis (n = 250), Müllerian duct anomalies (n = 167), endocrine disorders including diabetes and thyroid disorders (n = 174), or preimplantation genetic testing cycles (n = 481). After applying these exclusion criteria, 3986 patients were included in the final analysis.

### Baseline characteristics

As presented in Table 1, the study demonstrated that significant differences in baseline characteristics were observed between the good-quality (N = 3023) and poor-quality (N = 963) blastocyst groups prior to propensity score matching (PSM). The good-quality group had a higher proportion of younger women (< 30 years: 43.04% vs. 33.33%) and men (< 30 years: 37.16% vs. 26.06%), lower basal FSH levels (6.25 ± 1.50 vs. 6.54 ± 1.64 IU/L, *P* < 0.001), higher basal AMH levels (5.02 ± 3.08 vs. 4.07 ± 2.90 ng/mL, *P* < 0.001), and a greater proportion of primary infertility (43.86% vs. 32.50%, P < 0.001). Additionally, the good-quality group had a shorter infertility duration (≤ 2 years: 38.31% vs. 28.05%, *P* < 0.001), a higher proportion of primiparous women (68.38% vs. 54.41%, *P* < 0.001), and more conventional IVF cycles (73.77% vs. 69.57%, *P* = 0.011). Treatment-related parameters also differed, with the good-quality group having more hormone therapy cycles for endometrial preparation (67.85% vs. 57.63%, *P* < 0.001) and a higher proportion of day 5 embryo transfers (87.83% vs. 61.99%, *P* < 0.001). After PSM, 1,743 cycles were included (1,162 good-quality and 581 poor-quality blastocyst transfers), and baseline characteristics were well-balanced between the two groups, with no significant differences in maternal age (32.4 ± 4.0 vs. 32.5 ± 4.1 years, *P* = 0.4326), BMI (21.8 ± 3.1 vs. 21.9 ± 3.1 kg/m^2^, *P* = 0.5495), paternal age (*P* = 0.9133), basal FSH (6.40 ± 1.53 vs. 6.36 ± 1.51 IU/L, *P* = 0.6169), basal AMH (4.51 ± 2.76 vs. 4.50 ± 3.11 ng/mL, *P* = 0.9417), primary infertility (34.60% vs. 34.60%, *P* = 1.0000), infertility duration (≤ 2 years: 29.00% vs. 28.74%, *P* = 0.9553), or primiparity (55.77% vs. 56.97%, *P* = 0.6696). Treatment-related parameters, including the proportion of conventional IVF cycles (73.41% vs. 71.77%, *P* = 0.5051), hormone therapy cycles (60.93% vs. 60.07%, *P* = 0.7682), and day 5 embryo transfers (91.48% vs. 89.67%, *P* = 0.2514), were also comparable, indicating that PSM effectively minimized baseline differences between the two groups.

**Table 1:** Baseline characteristics of patients before and after propensity score matching.

	Before propensity score matching			After propensity score matching		
	GQE(N = 3023)	PQE(N = 963)	*P *value	GQE(N = 1162)	PQE(N = 581)	*P *value
Maternal age (years)			< 0.001			0.4326
˂ 30	1301 (43.04%)	321 (33.33%)		400 (34.43%)	215 (37.01%)	
30–35	1294 (42.81%)	416 (43.20%)		535 (46.04%)	249 (42.86%)	
≥ 35	428 (14.16%)	226 (23.47%)		227 (19.54%)	117 (20.14%)	
Maternal BMI (kg/m^2^)			0.721			0.5495
˂ 18.5	170 (5.63%)	47 (4.89%)		49 (4.22%)	30 (5.16%)	
18.5	2025 (67.03%)	639 (66.42%)		802 (69.02%)	388 (66.78%)	
25–30	700 (23.17%)	232 (24.12%)		253 (21.77%)	138 (23.75%)	
≥ 30	126 (4.17%)	44 (4.57%)		58 (4.99%)	25 (4.30%)	
Paternal age (years)			< 0.001			0.9133
˂ 30	1123 (37.16%)	251 (26.06%)		356 (30.64%)	176 (30.29%)	
30–35	1310 (43.35%)	442 (45.90%)		512 (44.06%)	262 (45.09%)	
≥ 35	589 (19.49%)	270 (28.04%)		294 (25.30%)	143 (24.61%)	
Paternal BMI (kg/m^2^)			0.954			0.4960
˂ 18.5	68 (2.38%)	20 (2.21%)		23 (1.98%)	12 (2.07%)	
18.5	1375 (48.03%)	443 (48.90%)		555 (47.76%)	263 (45.27%)	
25–30	1149 (40.13%)	361 (39.85%)		439 (37.78%)	235 (40.45%)	
≥ 30	271 (9.47%)	82 (9.05%)		116 (9.98%)	48 (8.26%)	
Basal FSH (IU/L)	6.25 ± 1.50	6.54 ± 1.64	< 0.001	6.40 ± 1.53	6.36 ± 1.51	0.6160
Basal AMH (ng/mL)	4.38[2.80, 6.56]	3.29[2.03, 5.29]	< 0.001	4.05[2.44,5.87]	3.69[2.24,5.87]	0.1920
Primary infertility	1326 (43.86%)	313 (32.50%)	< 0.001	402 (34.60%)	201 (34.60%)	1.0000
Infertility duration			< 0.001			0.9553
≤ 2 years	1149 (38.31%)	269 (28.05%)		337 (29.00%)	167 (28.74%)	
≥ 3 years	1850 (61.69%)	690 (71.95%)		825 (71.00%)	414 (71.26%)	
Primiparous (%)	2067 (68.38%)	524 (54.41%)	< 0.001	648 (55.77%)	331 (56.97%)	0.6696
Causes of infertility			0.45			0.1950
Tubal factor	1420 (46.97%)	445 (46.21%)		566 (48.71%)	272 (46.82%)	
PCOS	313 (10.35%)	87 (9.03%)		102 (8.78%)	65 (11.19%)	
Endometriosis	59 (1.95%)	27 (2.80%)		14 (1.21%)	12 (2.07%)	
Male factor	300 (9.92%)	92 (9.55%)		135 (11.62%)	52 (8.95%)	
Mixed factors	73 (2.41%)	22 (2.28%)		30 (2.58%)	15 (2.58%)	
Others	858 (28.38%)	290 (30.11%)		315 (27.11%)	165 (28.40%)	
FET cycle number			0.018			0.2967
1	2645 (87.50%)	814 (84.53%)		1022 (87.95%)	500 (86.06%)	
≥ 2	378 (12.50%)	149 (15.47%)		140 (12.05%)	81 (13.94%)	
Fertilization method			0.011			0.5051
IVF	2230 (73.77%)	670 (69.57%)		853 (73.41%)	417 (71.77%)	
ICSI	793 (26.23%)	293 (30.43%)		309 (26.59%)	164 (28.23%)	
FET endometrial preparation			< 0.001			0.7682
Natural cycle	972 (32.15%)	408 (42.37%)		454 (39.07%)	232 (39.93%)	
Hormone therapy cycle	2051 (67.85%)	555 (57.63%)		708 (60.93%)	349 (60.07%)	
Development stage at transfer			< 0.001			0.2514
Day 5	2655 (87.83%)	597 (61.99%)		1063 (91.48%)	521 (89.67%)	
Day 6	368 (12.17%)	366 (38.01%)		99 (8.52%)	60 (10.33%)	

GQE: good-quality embryo, PQE: poor-quality embryo. Continuous variables are expressed as mean ± standard deviation (SD) or medians [Q1–Q3], whereas categorical data are presented as n (%). For continuous variables, Welch’s t-test was used for FSH, whereas the Wilcoxon rank-sum test was used for AMH; categorical variables were compared using the chi-square test or Fisher’s exact test, as appropriate.

### Neonatal and obstetric outcomes

As shown in Table 2, after PSM, the incidence of congenital malformations was comparable between the good-quality (N = 1162) and poor-quality (N = 581) blastocyst groups (1.72% vs. 2.07%, P = 0.7525). Birth weight categories, including very low birth weight (VLBW, 0.60% vs. 0.86%, *P* = 0.7587), low birth weight (LBW, 3.10% vs. 3.96%, P = 0.4260), and high birth weight (HBW, 0.86% vs. 1.72%, *P* = 0.1765), were also similar. Other neonatal outcomes, such as mean gestational age at birth (37.89 ± 2.63 vs. 38.00 ± 2.51 weeks, *P* = 0.4025), birth weight (3407.32 ± 524.86 vs. 3442.86 ± 559.61 g, *P* = 0.2110), and birth length (50.39 ± 2.02 vs. 50.42 ± 2.12 cm, *P* = 0.8202), showed no significant differences. The rates of very small for gestational age (VSGA, 0.69% vs. 0.52%, *P* = 0.9148), small for gestational age (SGA, 2.75% vs. 3.10%, *P* = 0.7998), large for gestational age (LGA, 30.72% vs. 33.91%, *P* = 0.1966), and very large for gestational age (VLGA, 14.29% vs. 16.01%, *P* = 0.3784) were also comparable. NICU admission rates were extremely low, with no admissions in the good-quality group and only one case (0.17%) in the poor-quality group (*P* = 0.7236). Stillbirth rates were similarly low and showed no significant differences (0.34% vs. 0.52%, *P* = 0.8935).

**Table 2:** Neonatal and obstetric outcomes in good-quality and poor-quality embryo groups after propensity score matching.

Variables	GQE(N = 1162)	PQE(N = 581)	Standardized difference	*P *value
Gestational age at birth	38[38,39]	39[38,39]	0.0432	0.0892
Preterm birth	137 (11.79%)	58 (9.98%)	0.0606	0.2947
Newborn weight	3407.32 ± 524.86	3442.86 ± 559.61	0.0655	0.2110
Length at birth	50[50,51]	50[50,51]	0.0129	0.5103
Newborn gender (Male)	637 (54.82%)	306 (52.67%)	0.0459	0.3985
Cesarean delivery	902 (77.62%)	437 (75.22%)	0.0390	0.4819
Congenital malformation	20 (1.72%)	12 (2.07%)	0.0253	0.7525
Very low birth weight (VLBW)	7 (0.60%)	5 (0.86%)	0.0303	0.7587
Low birth weight (LBW)	36 (3.10%)	23 (3.96%)	0.0467	0.4260
High birth weight (HBW)	10 (0.86%)	10 (1.72%)	0.0763	0.1765
Very small for gestational age (VSGA)	8 (0.69%)	3 (0.52%)	0.0222	0.9148
Small for gestational age (SGA)	32 (2.75%)	18 (3.10%)	0.0204	0.7998
Large for gestational age (LGA)	357 (30.72%)	197 (33.91%)	0.0681	0.1966
Very large for gestational age (VLGA)	166 (14.29%)	93 (16.01%)	0.0480	0.3784
NICU admission	0 (0.00%)	1 (0.17%)	0.0587	0.7236
Stillbirth	4 (0.34%)	3 (0.52%)	0.0263	0.8935
Obstetric complications	146 (12.56%)	69 (11.88%)	0.0210	0.7378
Gestational hypertension	56 (4.82%)	18 (3.10%)	0.0884	0.1202
Gestational diabetes mellitus	35 (3.01%)	27 (4.65%)	0.0853	0.1095
Fetal distress	5 (0.43%)	0 (0.00%)	0.0930	0.2677
Amniotic fluid abnormalities	9 (0.77%)	3 (0.52%)	0.0322	0.7587
Premature rupture of membranes	40 (3.44%)	15 (2.58%)	0.0504	0.4102
Placenta previa	4 (0.34%)	6 (1.03%)	0.0833	0.1449
Placental implantation disease	0 (0.00%)	1 (0.17%)	0.0587	0.7236
Placental abruption	2 (0.17%)	0 (0.00%)	0.0587	0.8025
Cervical insufficiency	11 (0.95%)	2 (0.34%)	0.0753	0.2789

GQE: good-quality embryo, PQE: poor-quality embryo. Values are presented as mean ± SD or median [Q1–Q3] for continuous variables, and as n (%) for categorical variables. For continuous variables, Welch’s t-test was used for newborn weight; the Wilcoxon rank-sum test was used for gestational age at birth, and birth length; categorical variables were compared using the chi-square test or Fisher’s exact test, as appropriate.

Obstetric outcomes were also comparable between the two groups. The overall rate of obstetric complications (12.56% vs. 11.88%, *P* = 0.7378) and specific complications, including gestational hypertension (4.82% vs. 3.10%, *P* = 0.1202), gestational diabetes mellitus (3.01% vs. 4.65%, *P* = 0.1095), fetal distress (0.43% vs. 0.00%, *P* = 0.2677), and premature rupture of membranes (3.44% vs. 2.58%, *P* = 0.4102), showed no significant differences. These findings indicate that blastocyst quality was not associated with significant differences in neonatal or obstetric outcomes. Detailed test statistics for continuous outcomes after PSM are provided in Supplementary Table S2.

### Multivariate analysis of neonatal and obstetric outcomes

Multivariate analysis confirmed that blastocyst quality was not significantly associated with adverse neonatal or obstetric outcomes after adjusting for confounders (Table 3). For neonatal outcomes, no significant differences were observed in congenital malformations (adjusted OR = 1.14, 95% CI 0.54–2.41, *P* = 0.7310), preterm birth (adjusted OR = 0.80, 95% CI 0.57–1.12, *P* = 0.1976), or birth weight categories, including VLBW, LBW, and HBW (all P > 0.05). Obstetric outcomes, including mode of delivery (adjusted OR = 0.87, 95% CI 0.68–1.12, *P* = 0.2806) and overall complications (adjusted OR = 0.89, 95% CI 0.64–1.22, *P* = 0.4629), were also comparable.

**Table 3:** Multivariate logistic regression analysis of neonatal and obstetric outcomes between good-quality and poor-quality blastocyst groups.

	Non-adjusted		Adjusted	
	OR (95% CI)	*P *value	OR (95% CI)	*P *value
Congenital malformations	1.20 (0.58, 2.48)	0.6143	1.14 (0.54, 2.41)	0.7310
Neonatal gender	0.91 (0.75, 1.12)	0.3704	0.93 (0.75, 1.14)	0.4720
Preterm birth	0.83 (0.60, 1.15)	0.2597	0.80 (0.57, 1.12)	0.1976
VLBW (very low birth weight)	1.43 (0.45, 4.53)	0.5410	1.52 (0.47, 4.90)	0.4832
LBW (low birth weight)	1.29 (0.76, 2.20)	0.3502	1.26 (0.72, 2.19)	0.4172
HBW (high birth weight)	2.02 (0.83, 4.87)	0.1189	2.49 (0.94, 6.65)	0.0677
VSGA (very small for gestational age	0.75 (0.20, 2.83)	0.6699	0.77 (0.20, 2.98)	0.7053
SGA (small for gestational age)	1.13 (0.63, 2.03)	0.6850	1.10 (0.61, 1.99)	0.7461
LGA (large for gestational age)	1.16 (0.94, 1.43)	0.1785	1.17 (0.94, 1.45)	0.1691
VLGA (very large for gestational age)	1.14 (0.87, 1.51)	0.3412	1.14 (0.85, 1.52)	0.3832
Mode of delivery	0.91 (0.72, 1.16)	0.4446	0.87 (0.68, 1.12)	0.2806
Obstetric complications	0.94 (0.69, 1.27)	0.6803	0.89 (0.64, 1.22)	0.4629
Gestational hypertension	0.63 (0.37, 1.08)	0.0956	0.54 (0.30, 1.04)	0.0519
Gestational diabetes	1.57 (0.94, 2.62)	0.0847	1.46 (0.85, 2.50)	0.1660
Amniotic fluid abnormalities	0.66 (0.18, 2.47)	0.5417	0.77 (0.20, 2.94)	0.6980
Premature rupture of membranes	0.74 (0.41, 1.36)	0.3342	0.77 (0.42, 1.41)	0.3910
Placenta previa	3.02 (0.85, 10.75)	0.0877	2.94 (0.82, 10.61)	0.0990
Cervical insufficiency	0.36 (0.08, 1.64)	0.1865	0.42 (0.09, 1.99)	0.2764
Stillbirth	1.50 (0.34, 6.74)	0.5948	1.73 (0.37, 8.01)	0.4824

Values are reported as β (95% CI), *P *value, or OR (95% CI), *P *value. *P *values were calculated using the chi-square test. Multivariate logistic regression analysis was performed to assess the association between embryo quality and obstetric outcomes. The model was adjusted for fertilization method (IVF, ICSI), treatment regimen (natural cycle, hormone replacement cycle), embryo development stage (D5, D6), primiparous (yes, no), maternal age group (< 30, 30–35, ≥ 35), maternal BMI group (< 18.5, 18.5–25, 25–30, ≥ 30), paternal age group (< 30, 30–35, ≥ 35), and paternal BMI group (< 18.5, 18.5–25, 25–30, ≥ 30). Results are expressed as adjusted odds ratios (OR) with corresponding 95% confidence intervals (CI).

### Congenital malformation analysis

Detailed analysis of congenital malformations based on ICD-10 codes (Table 4) revealed no significant differences between the good-quality (N = 1162) and poor-quality (N = 581) blastocyst groups. The overall rate of congenital malformations was low and comparable (1.72% vs. 2.07%, *P* = 0.6138). Specific malformations, including congenital heart defects (Q21), spina bifida (Q05), and chromosomal abnormalities (Q90, Q99), occurred at very low frequencies, with no statistically significant differences (all *P* > 0.05). Rare malformations, such as congenital skin anomalies (Q82) and musculoskeletal anomalies (Q79), were observed only in the poor-quality group (0.34% each), but these differences were not statistically significant (*P* = 0.2110 for both). Cases of multiple malformations were extremely rare, with only one case (0.09%) in the good-quality group and none in the poor-quality group (*P* = 0.4794), further confirming that blastocyst quality was not associated with an increased risk of congenital malformations.

**Table 4:** Congenital malformation analysis based on ICD-10 codes.

ICD codes	GQE(N = 1162)	PQE(N = 581)	Chi-square	*P *value
Q05	1 (0.09%)	0 (0.00%)	0.5003	0.4794
Q17	1 (0.09%)	1 (0.17%)	0.2503	0.6169
Q21	5 (0.43%)	6 (1.03%)	2.2414	0.1344
Q37	1 (0.09%)	0 (0.00%)	0.5003	0.4794
Q42	1 (0.09%)	0 (0.00%)	0.5003	0.4794
Q43	2 (0.17%)	0 (0.00%)	1.0011	0.317
Q54	3 (0.26%)	0 (0.00%)	1.5026	0.2203
Q63	1 (0.09%)	0 (0.00%)	0.5003	0.4794
Q65	1 (0.09%)	0 (0.00%)	0.5003	0.4794
Q79	0 (0.00%)	2 (0.34%)	4.0046	0.2110
Q82	0 (0.00%)	2 (0.34%)	4.0046	0.2110
Q90	1 (0.09%)	1(0.17%)	0.2503	0.6169
Q99	2 (0.17%)	0 (0.00%)	1.0011	0.3170
Multiple congenital malformations	1 (0.09%)	0 (0.00%)	0.5003	0.4794
Congenital malformations	20 (1.72%)	12 (2.07%)	0.2547	0.6138

GQE: good-quality embryo, PQE: poor-quality embryo. Values are presented as n (%). Congenital malformations were classified according to ICD-10 codes. *P *values were calculated using Pearson’s Chi-squared test, with Yates’ continuity correction applied for cases with low expected frequencies.

## Discussion

In this propensity score-matched cohort study of 1,743 frozen-thawed single blastocyst transfer cycles, we found no significant difference in congenital malformation rates between good- and poor-quality embryo groups (1.72% vs. 2.07%, P = 0.7525). This finding remained consistent when congenital malformations were categorized by organ system according to ICD-10 codes, suggesting that poor embryo morphology alone may not be associated with an increased risk of birth defects.

Previous studies investigating the relationship between embryo quality and offspring safety are relatively limited. In a case–control study comparing 74 very poor quality (VPQ) embryos with 1507 top quality (TQ) embryos, Mendoza et al. demonstrated that VPQ embryo transfer was not associated with increased congenital malformations (1.35% vs. 1.72%) or perinatal complications^21^. A recent prospective study by Zhang et al. provided valuable long-term follow-up data, demonstrating that children aged 4–6 years conceived from poor-quality embryos exhibited comparable metabolic indicators and cognitive development to those from good-quality embryos in fresh cleavage-stage transfers^22^. Consistent findings were reported in several studies focusing on fresh embryo transfer cycles. Oron et al. analyzed 1541 fresh single embryo transfers and found no association between poor embryo quality and adverse obstetric or perinatal outcomes after adjusting for maternal variables^23^. Similarly, both Bouillon et al. and Akamie et al. demonstrated that single poor-quality blastocyst transfer did not adversely affect obstetric or perinatal outcomes compared to good-quality blastocysts^13,24^. Although these supporting studies were conducted in fresh transfer cycles rather than frozen-thawed embryo transfers as in our study, their findings collectively suggest the safety of poor-quality embryo transfers.

In contrast, some studies have reported different results. In a large-scale population-based registry study, Abel et al. reported that poor-quality embryos were associated with higher rates of abnormalities, particularly major anomalies and musculoskeletal abnormalities^25^. However, several methodological limitations warrant consideration when interpreting these results. First, the study design did not distinguish between fresh and frozen-thawed cycles in the analysis, and twin pregnancies were included in the study population. Second, data collection from multiple IVF centers (central, satellite and interstate clinics) may introduced potential inter-observer variations in embryo grading. Third, significant baseline differences existed between the study groups, including ICSI utilization rates, FET proportions, and treatment locations (P < 0.001). Additionally, the embryo grading was performed using an in-house system that did not separately assess trophectoderm and inner cell mass quality, which might have affected the accuracy of embryo quality classification and limited both the generalizability of their findings and the possibility for independent validation in other centers.

Birth weight outcomes have also been a focus of investigation in relation to embryo quality. Two independent studies reported an association between poor-quality embryos and reduced birth weight. Zhang et al. and Huang et al. both found that singletons born from poor-quality blastocysts had lower birth weights compared to those from good-quality blastocysts^26,27^. However, the interpretation of these findings requires careful consideration of their methodological limitations. Both studies enrolled relatively small cohorts (Huang 2020, n = 1306; Zhang 2020, n = 1207) and exhibited marked between-group imbalances in key baseline covariates, including day of embryo transfer (day 5 vs. day 6), year of treatment, and oocyte yield per cycle (total and MII oocytes). While Zhang et al. focused specifically on frozen–thawed transfers, they applied embryo-quality classification criteria that differ from those used in other reports, complicating direct comparisons. By contrast, our study retained a larger analytic sample after propensity-score matching (n = 1743) and achieved balance across these critical variables. In addition, the prior studies restricted outcomes to birth weight and did not interrogate the broader spectrum of perinatal endpoints, particularly congenital malformations. Finally, considering the research locations, investigative teams and periods of data collection, the datasets used in the two reports may partially overlap, limiting the independence of their findings and warranting caution in interpretation and cross-study comparison.

Several potential mechanisms might explain why poor embryo morphology alone does not necessarily translate to increased congenital malformation risks. First, embryo morphological assessment is primarily based on static observations at specific time points, which may not fully reflect the dynamic nature of embryonic development and the embryo’s intrinsic developmental potential^28,29^. Second, studies have shown that embryos possess remarkable developmental plasticity and compensatory mechanisms during early development. Poor morphological features such as fragmentation or irregular blastomere size may not necessarily indicate compromised genetic integrity or developmental potential^30^. Furthermore, the successful implantation of a poor-quality embryo might itself indicate the embryo’s inherent viability despite suboptimal morphological appearance^31^.

Several limitations of our study should be acknowledged. First, despite propensity score matching, unmeasured confounding factors might still exist. Second, our follow-up period was limited to birth outcomes and early infancy; therefore, long-term developmental outcomes remain unknown. Third, although our sample size was substantial, some rare congenital malformations might have been missed due to their extremely low incidence rates. Fourth, our study was conducted at a single center, potentially limiting the generalizability of our findings to other populations or centers using different embryo grading systems.

Future research directions should focus on several aspects. First, large-scale multicenter studies with standardized embryo grading systems are needed to validate our findings across different populations and clinical settings^32^. Second, long-term follow-up studies should be conducted to evaluate developmental, cognitive, and health outcomes beyond the perinatal period^33^. Third, the integration of time-lapse imaging and artificial intelligence-based assessment tools might provide more objective and comprehensive evaluation of embryo quality^34^. Finally, investigation of molecular and genetic markers in conjunction with morphological assessment might help better understand the relationship between embryo quality and developmental outcomes^35^.

## Conclusion

This study demonstrates that poor-quality embryo transfer does not significantly increase the risk of congenital malformations compared to good-quality embryo transfer. These findings provide reassuring evidence for the use of poor-quality embryos in cases where embryo selection is limited. However, given the limitations of this study, larger multicenter studies with longer follow-up periods are needed to validate these results further. Long-term assessment of offspring health and developmental outcomes remains a critical area for future investigation.

## Supplementary Information

Below is the link to the electronic supplementary material.

Supplementary Material 1

Supplementary Material 2

Supplementary Material 3

## Data Availability

The datasets used and analysed during the current study are available from the corresponding author on reasonable request.

## Abbreviations

ART: Assisted reproductive technology
AMH: Anti-Müllerian hormone
BMI: Body mass index
CI: Confidence interval
FET: Frozen embryo transfer
FSH: Follicle-stimulating hormone
GQE: Good-quality embryo
HBW: High birth weight
ICD: International classification of diseases
ICSI: Intracytoplasmic sperm injection
IVF: In vitro fertilization
LBW: Low birth weight
LGA: Large for gestational age
NICU: Neonatal intensive care unit
OR: Odds ratio
PCOS: Polycystic ovary syndrome
PQE: Poor-quality embryo
PSM: Propensity score matching
SD: Standard deviation
SGA: Small for gestational age
STROBE: Strengthening the reporting of observational studies in epidemiology
VLBW: Very low birth weight
VLGA: Very large for gestational age
VSGA: Very small for gestational age
VPQ: Very poor quality
TQ: Top quality
WHO: World Health Organization
