# Supplementary material for: Blastocyst quality and congenital malformation risk in singleton births after frozen embryo transfer
Source: Sci Rep. 2025 Oct 17;15:36326. doi: 10.1038/s41598-025-20150-2 (PMC12534368; doi:10.1038/s41598-025-20150-2)
Supplement: Supplementary file 1 — Supplementary Material 1 [file 41598_2025_20150_MOESM1_ESM.docx]

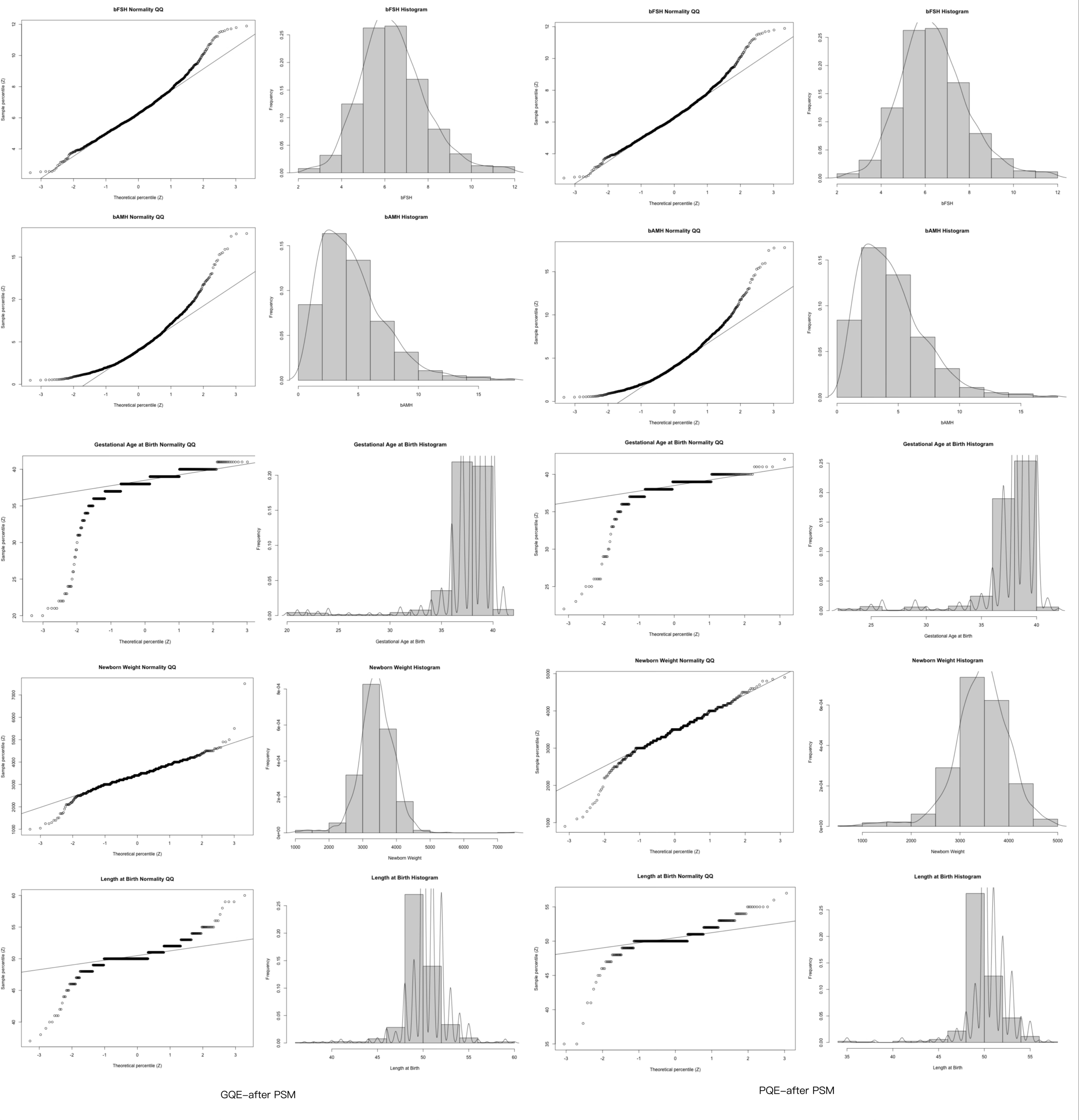


Supplementary Figure S1. Q–Q plots and histograms for continuous variables by group (GQE vs PQE) after PSM.
