# Supplementary material for: Blastocyst quality and congenital malformation risk in singleton births after frozen embryo transfer
Source: Sci Rep. 2025 Oct 17;15:36326. doi: 10.1038/s41598-025-20150-2 (PMC12534368; doi:10.1038/s41598-025-20150-2)
Supplement: Supplementary file 2 — Supplementary Material 2 [file 41598_2025_20150_MOESM2_ESM.docx]

| Supplementary Table S2. Comparisons of continuous variables after PSM: GQE (n=1162) vs PQE (n=581) | | | | | | | | | | | | |
| --- | --- | --- | --- | --- | --- | --- | --- | --- | --- | --- | --- | --- |
| Variable | Group | Mean | SD | Q1 | Median | Q3 | U(Mann–Whitney/Wilcoxon) | *P*-value (Mann–Whitney/Wilcoxon) | t(Welch) | *P*-value(Welch) | t(Student) | *P*-value(Student) |
| Basal FSH (IU/L) | GQE | 6.402 | 1.528 | 5.383 | 6.270 | 7.275 | 341889.5 | 0.662 | 0.502 | 0.616 | 0.500 | 0.617 |
|  | PQE | 6.364 | 1.515 | 5.370 | 6.260 | 7.270 |  |  |  |  |  |  |
| Basal AMH (ng/mL) | GQE | 4.513 | 2.756 | 2.443 | 4.045 | 5.870 | 350485.5 | 0.192 | 0.070 | 0.944 | 0.073 | 0.942 |
|  | PQE | 4.503 | 3.108 | 2.240 | 3.690 | 5.870 |  |  |  |  |  |  |
| Gestational age at birth (weeks) | GQE | 37.886 | 2.631 | 38 | 38 | 39 | 310330.5 | 0.089 | -0.851 | 0.395 | -0.837 | 0.403 |
|  | PQE | 37.997 | 2.511 | 38 | 39 | 39 |  |  |  |  |  |  |
| Newborn weight (g) | GQE | 3407.322 | 524.859 | 3100 | 3400 | 3750 | 296034.5 | 0.080 | -1.252 | 0.211 | -1.280 | 0.201 |
|  | PQE | 3442.855 | 559.609 | 3150 | 3500 | 3800 |  |  |  |  |  |  |
| Birth length (cm) | GQE | 50.389 | 2.015 | 50 | 50 | 51 | 209370.5 | 0.510 | -0.223 | 0.824 | -0.227 | 0.820 |
|  | PQE | 50.415 | 2.125 | 50 | 50 | 51 |  |  |  |  |  |  |
| Notes: Primary inference relied on the Wilcoxon rank-sum test and Welch t-tests; the Student t-test was provided as supplementary. Two-sided P-values are shown. | | | | | | | | | | | | |
