# Supplementary material for: Blastocyst quality and congenital malformation risk in singleton births after frozen embryo transfer
Source: Sci Rep. 2025 Oct 17;15:36326. doi: 10.1038/s41598-025-20150-2 (PMC12534368; doi:10.1038/s41598-025-20150-2)
Supplement: Supplementary file 3 — Supplementary Material 3 [file 41598_2025_20150_MOESM3_ESM.docx]

| Supplementary Table S1. Normality and variance homogeneity tests after PSM: GQE (n=1162) vs PQE (n=581) | | | | | | | | | | | |
| --- | --- | --- | --- | --- | --- | --- | --- | --- | --- | --- | --- |
| Method | Group | Basal FSH | | Basal AMH | | Gestational age at birth | | Newborn weight | | Birth length | |
|  |  | Statistic | *P*-value | Statistic | *P*-value | Statistic | *P*-value | Statistic | *P*-value | Statistic | *P*-value |
| Shapiro–Wilk test | GQE | 0.979 | <0.001 | 0.914 | <0.001 | 0.598 | <0.001 | 0.951 | <0.001 | 0.804 | <0.001 |
|  | PQE | 0.984 | <0.001 | 0.878 | <0.001 | 0.605 | <0.001 | 0.959 | <0.001 | 0.732 | <0.001 |
| Levene’s test | Overall | 0.0001 | 0.993 | 3.173 | 0.075 | 0.050 | 0.824 | 2.043 | 0.153 | 0.0003 | 0.986 |
| Notes:  Normality: Within-group normality was tested using the Shapiro–Wilk test; the reported Statistic is the Shapiro–Wilk W. Homoscedasticity: A modified robust Brown–Forsythe Levene-type test based on absolute deviations from the median (median-centered Levene) was used. The reported Statistic is the F value. The Levene row summarizes the combined two-group test (Group=Overall). Two-sided P-values are reported. | | | | | | | | | | | |
